# Understanding the Effects of Tensile Strain on the Structure and Magnetism of Stoichiometric LaCoO_3_ Films

**DOI:** 10.1021/acs.chemmater.5c03290

**Published:** 2026-03-12

**Authors:** Daniel Russell, Rebecca M. Haight, Binzhi Liu, Ali Barooni, Allen Partin, Alevtina Smekhova, Florian Kronast, L. Robert Baker, Maryam Ghazisaeidi, Jinwoo Hwang, Fengyuan Yang, Patrick M. Woodward

**Affiliations:** † Department of Physics, 2647 The Ohio State University, 191 W. Woodruff Avenue, Columbus, Ohio 43210, United States; ‡ Department of Chemistry and Biochemistry, 2647 The Ohio State University, 100 W. 18th Avenue, Columbus, Ohio 43210, United States; § Department of Materials Science and Engineering, 2647 The Ohio State University, 140 W. 19th Avenue, Columbus, Ohio 43210, United States; ∥ 28340 Helmholtz-Zentrum Berlin für Materialien und Energie, Albert-Einstein-Strasse 15, Berlin 12489, Germany

## Abstract

Despite numerous reports of an insulating ferromagnetic state in epitaxial LaCoO_3_ thin films, no consensus has been reached on the details of ferromagnetism in these films. To better understand the origins of magnetic order in such films, stoichiometric LaCoO_3_ films have been deposited on SrTiO_3_(001) and LaAlO_3_(001) substrates using off-axis sputtering. This technique allows growth to occur in conditions that minimize deviations from the ideal stoichiometry. SQUID magnetometry shows that ferromagnetism is stabilized only in films grown under tensile strain on SrTiO_3_. The magnetic properties of these films (*T*_C_ ≈ 70 K, *M*_sat_ ≈ 0.3 μ_B_/Co, and *H*_C_ ≈ 5 kOe) are essentially independent of thickness, consistent with nearly uniform magnetization. At room temperature, strain induced by the SrTiO_3_ substrate breaks the rhombohedral symmetry of the bulk structure, leading to *a* ^–^ *a* ^–^ *c* ^0^ octahedral tilting and an anisotropic distortion of the Co-centered octahedra. Low-temperature (*T* = 36 K) X-ray absorption spectroscopy reveals that tensile strain inherent to the SrTiO_3_ substrate stabilizes a substantial fraction of high- or intermediate-spin Co^3+^ ions, facilitating magnetic order, whereas films grown on LaAlO_3_ are made up nearly entirely of low-spin Co^3+^ ions.

## Graphical abstract

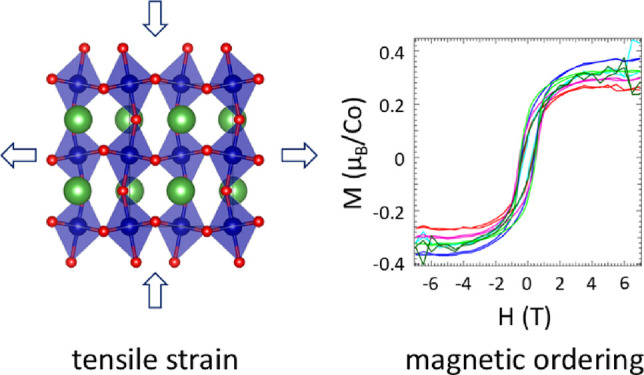

## 1 Introduction

Among oxide perovskites, few compounds have been more intensively studied than LaCoO_3_. Interest in this compound is largely driven by the fact that Hund’s coupling, the energy scale associated with intra-atomic exchange, is comparable to the octahedral crystal field splitting of the d-orbitals of Co^3+^. Hund’s coupling favors electrons populating different orbitals with parallel spin, and therefore a high-spin (HS) configuration for Co^3+^ (*t*_2g_^4^ *e*_g_^2^, *S* = 2), whereas crystal field splitting favors complete occupation of the *t*_2g_ orbitals, and therefore a nonmagnetic low-spin (LS) configuration (*t*_2g_^6^ *e*_g_^0^, S = 0). The competition between these two states is such that the mixture of HS and LS Co^3+^ ions is sensitive to external perturbations, including temperature, pressure, and epitaxial strain.^1,2^

The effects of temperature on the electronic, magnetic, and transport properties of bulk LaCoO_3_ have been extensively studied since the 1950s. The classic interpretation, first proposed by Goodenough, is that at low temperatures (*T* < 35 K) all Co^3+^ ions are in the LS state, save those ions near the surface, giving rise to a diamagnetic ground state.^3^ As the temperature increases the concentration of HS Co^3+^ rapidly increases at the expense of LS Co^3+^ until a 50:50 mixture of the two spin states is reached near 110 K. This situation persists until ∼350 K after which LS Co^3+^ ions are converted to intermediate-spin (IS) ions (*t*_2g_^5^ *e*_g_^1^, *S* = 1) and the *e*_g_ electrons start to delocalize. At still higher temperatures (*T* > 650 K) a homogeneous metallic state emerges.^4^ Subsequent studies tend to coalesce around a picture where all three spin states—HS, IS, LS—play a role in the temperature dependent magnetic and transport properties.^5,6^ Nevertheless, the exact nature of the spin state transitions remains under debate, as some recent studies have suggested IS Co^3+^ does not play a prominent role and the spin states evolve directly from LS to HS.^7^

Long range magnetic order is not observed in stoichiometric bulk samples. However, subtle perturbations that stabilize either HS or IS Co^3+^ ions at low temperatures can lead to a poorly understood ferromagnetic state. Weak ferromagnetism with a T_C_ ≈ 85 K is well documented to occur in some bulk samples, even single crystals.^8^ This behavior is attributed to regions near the surface, where the coordination of cobalt ions is reduced to either tetrahedral or square pyramidal, thereby stabilizing the HS state. However, neither the details of the crystal structure nor the magnetic structure associated with the ferromagnetic regions are understood.

Even more intriguing is the stabilization of a “ferromagnetic” insulating state in epitaxial films grown on substrates that induce tensile strain. Since the initial discovery in 2007,^9^ this behavior has been reported by many different groups.^10–27^ LaCoO_3_ films grown under 1–2% tensile strain exhibit Curie temperatures (*T*_C_) of 80–85 K, saturation magnetization (*M*_sat_) values of 0.5–1.2 μ_B_/Co, and coercive fields (*H*_C_) of ∼5 kOe. Not surprisingly there are variations in properties from one study to another, with some reports show nonsaturating magnetization approaching 2 μ_B_/Co.^28,29^ A summary of the properties previously reported for LaCoO_3_ films can be found in the Supporting Information (Table S1).

While there is agreement that stabilization of a ferromagnetic insulating state requires a mixture of spin states, there is no consensus on the details of the magnetic ground state. Some researchers propose that ferromagnetism is stabilized by strain, others by defects, and still others by a combination of the two. Many studies of epitaxial LaCoO_3_ films report periodically repeating dark stripes in high-resolution transmission electron microscopy (HRTEM) images. Some early studies attributed these to extreme modulations of the Co–O bond distances that arise from stripe-like ordering of HS and LS Co^3+^ ions, but spatially resolved TEM imaging and electron energy loss spectroscopy show that the dark stripes arise from the presence of ordered oxygen vacancies.^17,18^ Such defects create HS Co^2+^ ions (*t*_2g_^5^ *e*_g_^2^, *S* = 3/2) which can play a pivotal role in stabilizing magnetic ordering. The similarity in the Curie temperatures of films grown under tensile strain (80–85 K) with the surface ferromagnetism seen in bulk samples (≈85 K) raises the possibility that oxygen vacancies might be playing an important role in the magnetism seen in thin films. In fact, some studies conclude that both strain and oxygen vacancy ordering act synergistically to stabilize ferromagnetism in films grown under tensile strain.^11,12^

The overwhelming majority of LaCoO_3_ films studied to date have been grown using pulsed laser deposition (PLD). In a typical PLD growth the gas pressure is ∼150 mTorr and the substrate temperature is on the order of ∼700 °C. Both conditions are potentially problematic if one aspires to minimize deviations from the ideal stoichiometry. In those studies where the pressure inside the PLD chamber is varied, higher pressures lead to La-deficiencies on the order of 10%.^19,20^ The relatively high temperatures needed for PLD growth of LaCoO_3_ films may be even more problematic. High temperatures favor the evolution of molecular oxygen and the reduction of Co^3+^ to Co^2+^. In-situ neutron powder diffraction studies of bulk LaCoO_3_ in a dynamic vacuum show that oxygen vacancy concentrations start to increase when the sample is heated above 525 °C, leading to the partial reduction of Co^3+^ to Co^2+^.^6^ While not identical to conditions used in thin film growth, there is good reason to believe that the conditions used for PLD growth encourage the formation of oxygen vacancies, leading to the presence of dark stripes seen in so many studies of LaCoO_3_ films.

In this paper, we utilize off-axis sputtering^30^ to grow LaCoO_3_ films on single crystal substrates that induce both compressive (LaAlO_3_) and tensile (SrTiO_3_) strain. Depositions are carried out at pressures (∼12 mTorr) and temperatures (∼450 °C) that are much lower than previous studies. By doing so we seek to minimize deviations from the ideal LaCoO_3_ stoichiometry and thereby disentangle the role of strain and defects on the magnetism of LaCoO_3_ films. This allows us to answer several questions about the intriguing and poorly understood magnetism of near-stoichiometric LaCoO_3_ films. For example, what pattern of octahedral tilting is induced by tensile strain? How do the Co–O bond distances and Co–O–Co bond angles respond to epitaxial strain, particularly tensile strain? How does strain impact the distribution of Co spin states? Can ferromagnetism be stabilized by strain alone and if so, how does it differ from the combined effects of strain and oxygen vacancies? Is the magnetically ordered state a simple colinear ferromagnet or is it a more complicated pattern of ordered spins?

## 2 Experimental Section

A polycrystalline LaCoO_3_ target was synthesized using conventional solid-state synthesis techniques. Stoichiometric amounts of La_2_O_3_ and Co_3_O_4_ were ball milled and then heated to 1150 °C for 48 h. The La_2_O_3_ was previously dried at 1000 °C for 12 h to prevent hydration. Phase purity was confirmed through X-ray powder diffraction (XRPD) on a Bruker D8 Advance powder diffractometer (40 kV, 40 mA, sealed Cu X-ray tube) equipped with a Lynxeye XE-T position-sensitive detector. The diffractometer is configured with an incident beam monochromator (Johansson type SiO_2_-crystal) that selects only Cu Kα_1_ radiation (λ = 1.5406 Å). The XRPD pattern of the polycrystalline target is shown in the Supporting Information (Figure S1). After pressing, the target was sintered at a temperature of 1108 °C for 2 h.

LaCoO_3_ thin film samples were grown by off-axis RF-sputtering. The operating pressure was fixed at 12 mTorr with an atmosphere that was 93% Ar and 7% O_2_. The optimal temperature window for growth was narrow and dependent on the choice of substrate. Films grown on SrTiO_3_(001) grew optimally at 450 °C while the optimal growth temperature on LaAlO_3_ substrates was 475 °C. The films were cooled from the growth temperature to room temperature at a rate of 20 °C/min in the same atmosphere used for deposition. X-ray diffraction (XRD) data on epitaxial films were taken using a Rigaku SmartLab operating in parallel beam mode with a Ge(220) double bounce monochromator. Samples were aligned to the (002) peak of the substrate, and symmetric 2θ-ω scans were performed around this range. Rocking curve measurements were performed using a Panalytical Empyreon with a Ge(220) four bounce monochromator combined with a triple bounce analyzer to achieve maximum angular resolution. Reciprocal space mapping (RSM) was performed with off-symmetric 2θ-ω scans around the (103) substrate peak using a Ge(220) four bounce monochromator and a two-dimensional detector. The thicknesses of the films were determined via X-ray reflectivity (XRR) measurements carried out with a Panalytical Empyreon and the data were fit to simulations to confirm thickness.

Density functional theory (DFT) calculations were performed using the Vienna Ab initio Simulation Package (VASP), with the Projector Augmented Wave (PAW) method.^31,32^ The exchange-correlation functional was treated within the meta-generalized gradient approximation (meta-GGA) using the Regularized-Restored Strongly Constrained and Appropriately Normed (r^2^SCAN) formulation.^33^ A plane-wave energy cutoff of 550 eV was used, and the Brillouin zone was sampled using a gamma k-point spacing of 0.25 Å^–1^.

Scanning transmission electron microscopy was conducted to investigate the atomic structure with a probe-corrected Thermo Scientific Themis-Z STEM operated at 300 kV. The TEM thin foil was prepared via a FEI Helios NanoLab 600 DualBeam focus ion beam system. Nanodiffraction was performed using the Thermo Scientific Electron Microscope Pixel Array Detector.

Magnetization measurements on epitaxial films were carried out using a QuantumDesign MPMS3 operating in VSM mode. Samples were mounted for in-plane (IP) measurements with varnish and a quartz rod. Resistivity measurements were performed in a Dynacool Physical Property Measurement System (PPMS) from Quantum Design. Four-probe electrical measurements were carried out using a Keithley Model 2400 DC current source and a Keithley Model 2182 nanovoltmeter. The sample was patterned into a 10 × 40 μm bar, and a sensing current of 0.1 μA was applied.

X-ray absorption spectroscopy (XAS) measurements were performed at the SPEEM end-station of the UE49-PGMa beamline of BESSY II synchrotron radiation facility operated by Helmholtz-Zentrum-Berlin. Spectra of the Co L-edges were taken at 36 K to assess the spin states of cobalt at temperatures where magnetic ordering occurs (in films grown under tensile strain). Samples were capped with 0.5 nm platinum layer to prevent charging effects.

## 3 Results and Discussion

Epitaxial films of LaCoO_3_ with varying thicknesses were grown by off-axis sputtering on two perovskite substrates: SrTiO_3_ and LaAlO_3_. Substrate crystals were cut along the (001) face for a cube-on-cube growth. When grown on SrTiO_3_(001), LaCoO_3_ is under 2.0% tensile strain, whereas films grown on LaAlO_3_(001) are under 1.1% compressive strain. The XRD patterns of LaCoO_3_ films with various thicknesses grown on SrTiO_3_ can be seen in Figure 1a, with the rocking curve of the 35 nm LaCoO_3_ film shown in the inset. The full-width at half-maximum (fwhm) of the rocking curve, 0.0026°, is the narrowest reported to date for epitaxial LaCoO_3_ thin films.^18,22,23,29,34^ Figure 1b shows similar results for films grown on LaAlO_3_. All films showed numerous Laue oscillations, indicating high quality crystal growths. The out-of-plane (OOP) lattice parameters for the films are plotted in Figure S2. The OOP lattice parameters qualitatively respond to strain as expected for both substrates. The tensile strain of the SrTiO_3_ substrate leads to a contraction of the OOP lattice parameter, while the compressive strain of the LaAlO_3_ substrate has the opposite effect. The films grown on SrTiO_3_ appear to be fully strained up to a thickness of 35 nm. For the films of 50, 60, and 80 nm thickness a small degree of strain relaxation can be observed, as indicated by the subtle increase of the OOP lattice parameter. The films grown on LaAlO_3_ do not show any obvious trend. The OOP lattice parameter averages to 3.869 Å.

**Figure 1:**
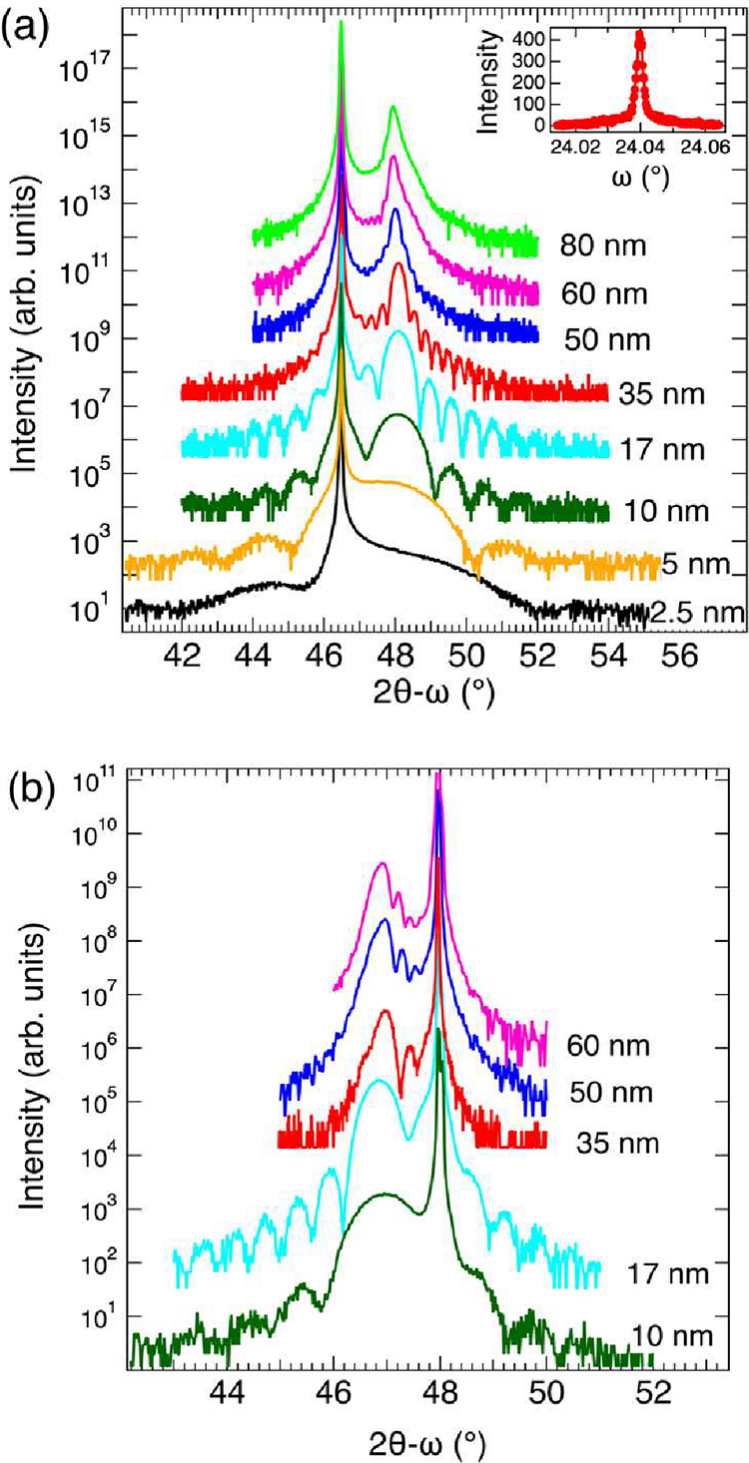
X-ray diffraction patterns of LaCoO_3_ films of various thicknesses grown on (a) SrTiO_3_(001) and (b) LaAlO_3_(001) substrates.

X-ray reciprocal space mapping (RSM) characterizes the distribution of scattered intensity in reciprocal space. Bragg peaks can determine crystal structure while satellite peaks and diffuse scattering indicate the presence of superlattices or in plane structural modulations.^23,34^ RSM shown in the Supporting Information (Figure S3) confirm that our films are fully strained to the substrate. Scans along the pseudocubic [103] axis show no streaking or smearing of the film intensity relative to that of the SrTiO_3_ substrate in either the IP or OOP directions.

Experimental IP and OOP lattice parameters of the strained films are compared to the bulk in Table 1. Given the RSM results the IP lattice parameters are assumed to be equal to that of the substrate. Films under tensile strain from the underlying SrTiO_3_ substrate exhibit a unit cell volume (57.64 Å^3^) that is 2.7% larger than bulk LaCoO_3_, while films grown under compressive strain on LaAlO_3_ have a unit cell volume (55.56 Å^3^) that is 1.0% smaller. A more nuanced analysis using the Poisson ratio (estimated to be ν = 1/3 for LaCoO_3_) can be used to predict the OOP lattice parameter.^35^ Using this approach we see good agreement between the observed and expected OOP lattice parameter for films grown on LaAlO_3_, but the observed OOP lattice parameter for films grown on SrTiO_3_ (3.780 Å) is larger than predicted (3.753 Å). The anomalously large value of the OOP lattice parameter and unit cell volume for LaCoO_3_ films grown under tensile strain could be signaling an increase in the concentration of HS Co^3+^ (*r* = 0.61 Å) relative to the smaller LS Co^3+^ (*r* = 0.54 Å).^36^

**Table 1:** In-Plane (IP) and Out-of-Plane (OOP) Lattice Parameters for 17 nm Thick LaCoO_3_ Films Grown under Tensile Strain on SrTiO_3_ and Compressive Strain on LaAlO_3_ ^a^

	bulk^b^	epitaxial on LaAlO_3_(001)	epitaxial on SrTiO_3_(001)
IP lattice parameter (Å)	3.829	3.787	3.905
OOP lattice parameter (Å)	3.829	3.874	3.780
unit cell volume (Å^3^)	56.14	55.56	57.64
Predicted OOP lattice parameter (Å)		3.871	3.753

a The predicted value of the OOP lattice parameter is calculated assuming a Poisson ratio ν = 1/3 given in reference 35.

b The bulk lattice parameters represent a pseudocubic unit cell. A cubic unit cell with the same volume as the rhombohedral unit cell at 300 K. Values for the rhombohedral unit cell were taken from ref 6.

Next, we turn to the electrical transport and magnetic properties of the films. The insulating nature of the films was confirmed with temperature-dependent resistance measurements (Figure S4). Even when applying a current of 0.1 μA, the resistance below 180 K exceeded the limit of the instrument and could not accurately be measured. The magnetization vs applied field (M vs H) scans show that LaCoO_3_ films grown on SrTiO_3_(001) are clearly ferromagnetic at 5 K, as shown in Figure 2a. In contrast, the lack of either coercivity or remnant magnetization in the LaCoO_3_ films grown on LaAlO_3_(001) signal the absence of ferromagnetism, as shown in Figure S5. The nonlinear response and upturn in magnetization seen at low temperatures is likely the result of magnetic impurities in the LaAlO_3_ substrate. For the films grown on SrTiO_3_(001) the saturation magnetization is on the order of 0.3 μ_B_ per Co ion and the coercivity varies from 5–6 kOe for thinner films, increasing slightly for thicker films. The magnetization vs temperature (M vs T) scans show Curie temperatures ranging from 63 to 70 K (Figure 2b). Full details of the magnetism of each film grown on SrTiO_3_(001) are given in the Supporting Information (Table S2).

**Figure 2:**
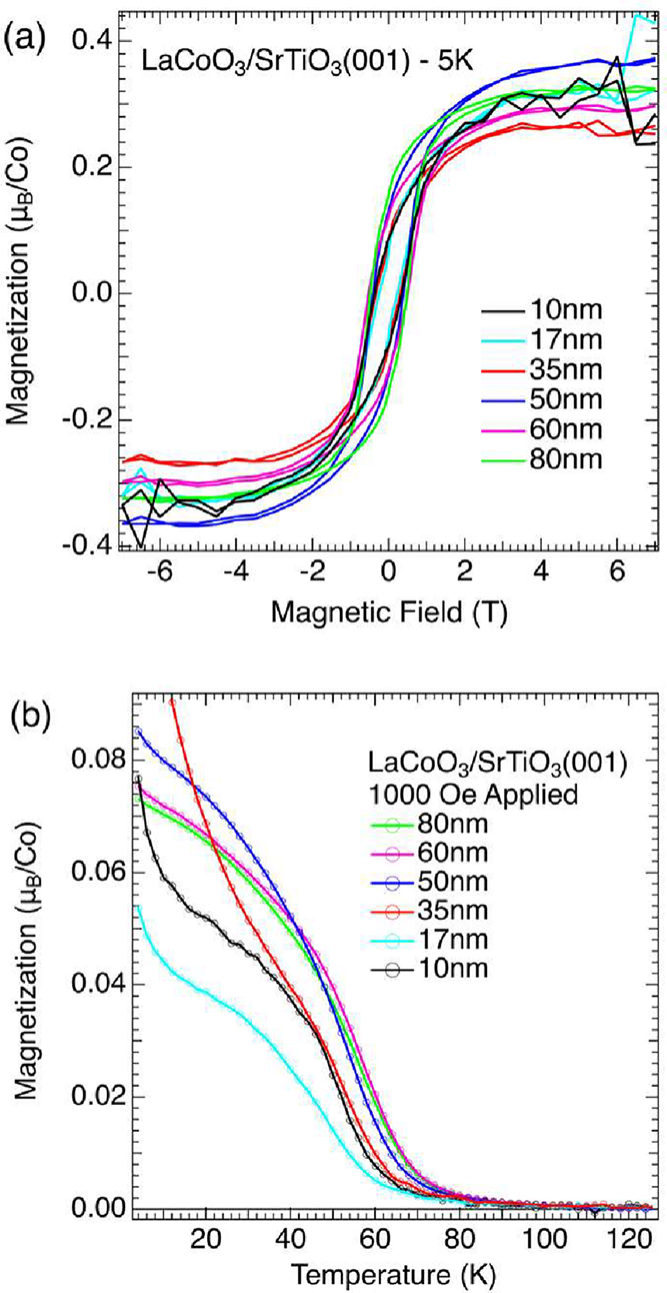
(a) Magnetization vs applied field for various LaCoO_3_ films grown on SrTiO_3_(001). (b) Magnetization vs temperature for the same films.

The ferromagnetism seen in LaCoO_3_ films grown on SrTiO_3_ is broadly in agreement with prior studies (T_C_ ≈ 85 K, H_C_ ≈ 5 kOe, and M_sat_ ≈ 1 μ_B_/Co), though there are some notable differences. The ordering temperatures of our films are somewhat lower and the saturation magnetization is smaller by a factor of ∼3 with respect to most earlier studies. It is also noteworthy that the saturation magnetization, normalized per Co ion, does not vary systematically with changes in film thickness. This result excludes the possibility of ferromagnetism being confined to the interface or the free surface. Hence, we can rule out the possibility that the ferromagnetism seen here is the same as what is seen on the surfaces of bulk samples. Taken together with the minimal structural relaxation discussed above, the data strongly suggest that the observed ferromagnetism is relatively uniform throughout the film and driven primarily by the tensile strain imposed by the SrTiO_3_ substrate. Given our interest in understanding the ferromagnetic state, we focus primarily on films grown on SrTiO_3_ for the rest of this paper.

To assess the effect of strain on key bond distances and bond angles we turn to constrained DFT calculations. Bulk LaCoO_3_ has out-of-phase rotations/tilts of the octahedra about all three axes of the pseudocubic unit cell, which lower the symmetry from cubic $Pm\overset{-}{3}m$ to rhombohedral $R\overset{-}{3}c$ . This pattern of octahedral tilting, denoted as *a* ^–^ *a* ^–^ *a* ^–^, can also be thought of as a single tilt about the cubic [111] axis.^37–39^ The imposition of epitaxial strain breaks the degeneracy of the tilts about the pseudocubic axes and will necessarily lead to a change in the pattern of octahedral tilting. If tilting about the axis perpendicular to the substrate remains out-of-phase the tilt system becomes *a* ^–^ *a* ^–^ *c* ^–^ (space group *C*2/*c*). If it goes to zero the tilt system becomes *a* ^–^ *a* ^–^ *c* ^0^ (space group *Imma*). If it switches over to in-phase tilting the tilt system becomes *a* ^–^ *a* ^–^ *c* ^+^ (space group *Pnma*). Chaturvedi et al. carried out DFT calculations to compare the energies of various patterns of octahedral tilting as a function of epitaxial strain.^23^ They found that all three of the above tilt systems are nearly degenerate when the tensile strain exceeds ∼1%. They went onto conclude that *a* ^–^ *a* ^–^ *c* ^+^ tilting and *Pnma* symmetry is the most stable pattern for films grown on SrTiO_3_ (2.0% tensile strain) but conceded that other tilt systems could easily be stabilized at finite temperatures.

To explore the effects of tensile strain on the crystal structure, we started from a 2a_p_ × 2a_p_ × 2a_p_ pseudocubic unit cell with atomic coordinates that correspond to the bulk rhombohedral structure and lattice parameters that are constrained in the *ab*-plane to match those of SrTiO_3_ (*a* = *b* = 2 × 3.905 Å = 7.810 Å), while the out-of-plane lattice constant and atomic coordinates were relaxed until the Hellman–Feynman on all atoms were reduced below 10 meV/Å. No symmetry other than translational symmetry was imposed on the structure. Non–spin-polarized calculations were chosen to focus on the coupling between the crystal structure, electronic structure, and epitaxial strain without the influence of spin degrees of freedom, as no long-range spin state ordering is expected near room temperature.

The geometry optimized structure is shown in Figure 3. The *c*-axis lattice parameter was optimized to a value of 7.511 Å, which reduces to 3.755 Å when converted to the smaller pseudocubic unit cell. This value is very close to the value of 3.753 Å predicted using Poisson ratio, but somewhat smaller than the 3.78 Å value seen experimentally. Nevertheless, it does qualitatively capture the contraction of the structure in the direction perpendicular to the substrate. A closer look at Figure 3 reveals out-of-phase tilts about both the *a*- and *b*-axes, and no tilting around the *c*-axis, corresponding to the *a* ^–^ *a* ^–^ *c* ^0^ pattern of tilting. If we perform a symmetry reduction the structure can be written with *Ibmm* symmetry (a nonstandard setting of *Imma*) and a unit cell that is half the volume of the 2a_p_ × 2a_p_ × 2a_p_ unit cell (Table S3).

**Figure 3:**
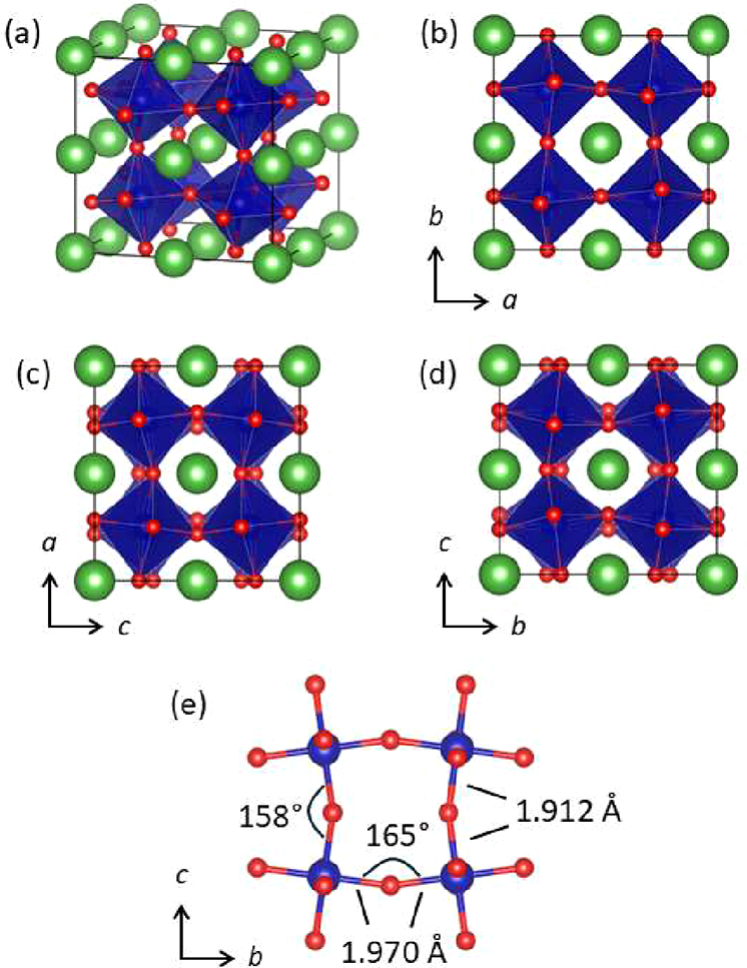
DFT-optimized structure of LaCoO_3_ grown in tensile strain on SrTiO_3_(001). (a) Perspective view with La, Co, and O shown as green, blue, and red spheres, respectively, viewed looking down the (b) *c*-axis showing the absence of octahedral tilts, (c) the *b*-axis showing out-of-phase tilts, and (d) the *a*-axis showing out-of-phase tilts. (e) An illustration of Co–O distances and Co–O–Co angles.

The pertinent bond distances and angles are shown in Figure 3e. Here we can see the effects of the tensile strain on the structure. Compared to the bulk values for the Co–O distance (1.935 Å) and Co–O–Co angle (163.7°), we see that the in-plane Co–O bonds have expanded (1.970 Å) and the Co–O–Co angle has slightly increased (165°). In contrast, the out-of-plane Co–O bonds have contracted (1.912 Å) and the Co–O–Co angle has become more acute (158°). This structure reveals the microscopic changes that lead to the observed in-plane expansion and out-of-plane contraction of the unit cell. In hindsight, the lack of tilting around the *c*-axis is not surprising. Such tilting, either in-phase or out-of-phase, would effectively decrease the lattice parameters in the *ab*-plane, which would exacerbate the mismatch with the SrTiO_3_ substrate.

High-resolution STEM was employed to investigate the effects of tensile strain on the atomic structure of LaCoO_3_. Figure 4a presents a high-angle annular dark field (HAADF) micrograph of the 35 nm-thick LaCoO_3_ film grown on the SrTiO_3_(001) substrate, viewed along the [100]_c_ zone axis (indexed in the pseudocubic system). The micrograph reveals a remarkably sharp and coherent heterointerface between the film and substrate, with no observable interfacial secondary phases or structural defects. Thus, demonstrating the excellent crystalline quality and epitaxial registry of the film grown using our optimized off-axis RF-sputtering technique. The uniform atomic arrangement within the LaCoO_3_ layer further confirms the high structural integrity and reproducibility of the growth process.

**Figure 4:**
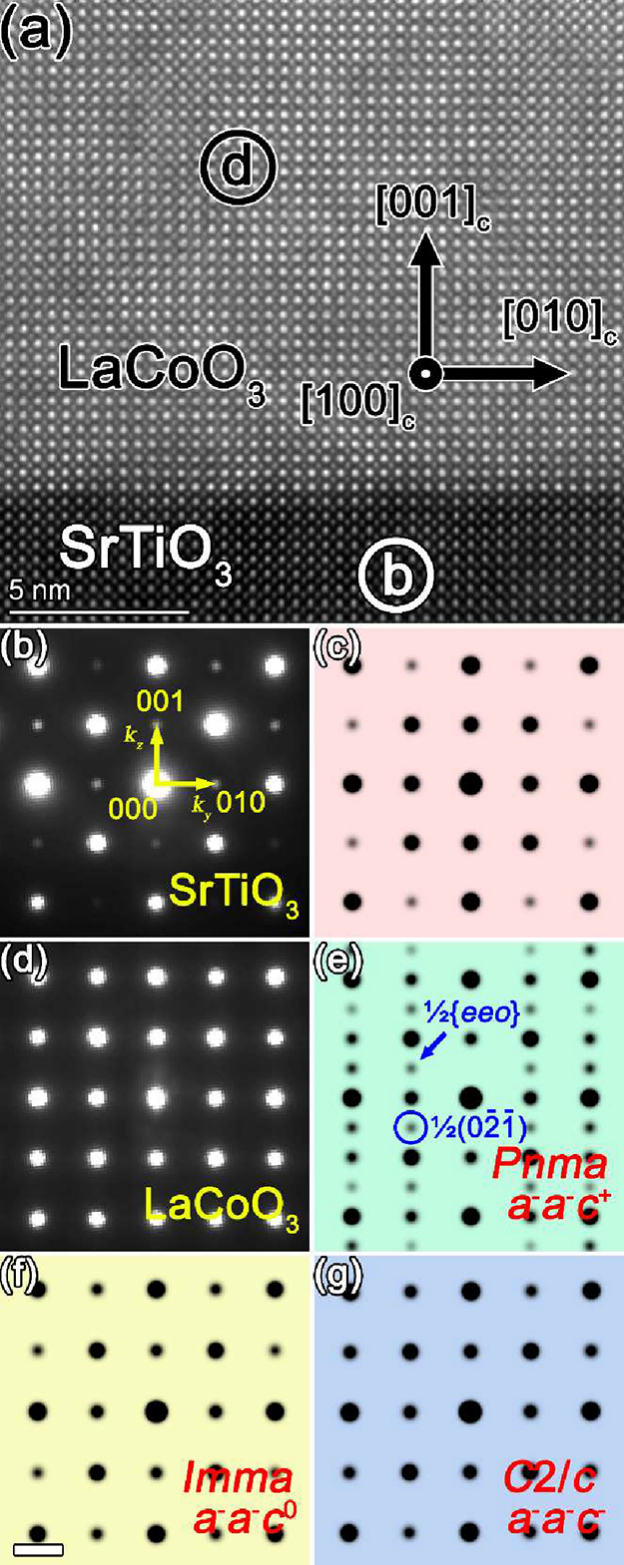
(a) HAADF micrograph of the LaCoO_3_(35 nm)/SrTiO_3_(001) film viewed from the [100]_c_-zone axis, indexed with a pseudocubic unit cell. Nanodiffraction patterns were acquired from the circled regions. Area b corresponds to SrTiO_3_ and area d to LaCoO_3_. The measured ratio of distances, *k*_*z*_ */k*_*y*_, between the (001) and (010) reflections relative to the direct reflection (000), is 1.001, for SrTiO_3_ and 1.029 for LaCoO_3_. (c) The simulated SrTiO_3_ diffraction pattern, yielding theoretical *k*_*z*_ */k*_*y*_ ratio of 1.000. (e–g) Simulated LaCoO_3_ diffraction patterns for structures with (e) *Pnma*, (f) *Imma*, and (g) *C*2*/c* symmetry. The scales in Figure 4b–g are identical; for simplicity, one scale bar corresponding to 2 nm^–1^ is shown in Figure 4f.

In contrast to previous reports on LaCoO_3_ films fabricated by high-pressure PLD, where vertical “dark stripe” features are often observed along the [100]_c_ zone axis,^17,18^ our sputtered films do not exhibit such stripes, consistent with a stoichiometric composition and the absence of long-range ordered defects. This observation underscores a key advantage of the RF-sputtering approach, which provides control over the stoichiometry that is superior to PLD, at least for the growth of LaCoO_3_ films.

To further elucidate the precise crystal symmetry, nanodiffraction experiments were subsequently performed on Area b (SrTiO_3_ substrate) and Area d (LaCoO_3_ film) in Figure 4a. The resulting experimental diffraction patterns, shown in Figure 4b,d, are systematically compared with simulated patterns in Figure 4c,e–g, representing SrTiO_3_ and potential LaCoO_3_ structures with *Imma*, *Pnma*, and *C*2/*c* space group symmetry, all viewed along the [100]_c_-zone axis. The *Imma* structure was obtained from the DFT calculations discussed above. The *Pnma* structure was obtained using the structure prediction software SPuDS^40^ with lattice parameters adjusted to match the *Imma* structure, and the *C*2/*c* structure was taken from the literature.^41^ The comparison between the simulated and experimental patterns was conducted in terms of ratio of reciprocal spacings, *k*_*z*_ */k*_*y*_, between the (001) and (010) reflections relative to the central (000) transmitted beam. The experimental value for the SrTiO_3_ substrate, 1.001, agrees exceptionally well with the expected value of 1.000 for a cubic perovskite, validating the reliability and accuracy of our diffraction analysis. The measured *k*_*z*_ */k*_*y*_ ratio of 1.029 for LaCoO_3_ closely matches the value of 1.032 derived from the DFT calculations for the orthorhombic *Imma* space group (*a* ^–^ *a* ^–^ *c* ^0^ tilt system), and the *k*_*z*_ */k*_*y*_ ratio of 1.033 calculated from the XRD lattice parameters. (Note that the reciprocal space *k*_*z*_ */k*_*y*_ is just the inverse of the real space *c*/*b*.)

The alternative orthorhombic structure with *Pnma* symmetry and *a* ^*–*^ *a* ^*–*^ *c* ^*+*^ tilting, is differentiated from the *Imma* structure by 12 ­{*eeo*} (even, even, odd)-type superlattice reflections arising from in-phase octahedral rotations along the *c*-axis (Figure 4e).^42^ Crucially, such reflections are not observed in the experimental diffraction pattern, Figure 4d, thereby allows us to exclude this space group from consideration. Although the electron diffraction patterns of the *Imma* (*a* ^–^ *a* ^–^ *c* ^0^ tilts) and *C*2/*c* (*a* ^–^ *a* ^–^ *c* ^–^ tilts) structures have the similar diffraction spots in this projection, other factors argue in favor of adoption of the former tilt system. First, out-of-phase tilts about the axis perpendicular to the substrate are allowed in our DFT calculations, but the geometry optimized structure indicates that those tilts go to zero. Second, if there were substantial tilts about the *c*-axis it would reduce the length of the *a* and *b* lattice constants pushing the *c*/*b* (and *c*/*a*) ratio toward unity, contrary to the findings of both electron and X-ray diffraction measurements.

Further unambiguous confirmation of the *Imma* symmetry was obtained through integrated differential phase contrast (iDPC) imaging, as shown in Figure 5. The LaCoO_3_/SrTiO_3_(001) film was viewed along the [110]_c_-zone axis, and HAADF and iDPC micrographs were acquired simultaneously. Owing to their distinct contrast mechanisms (Z-contrast for HAADF and nearly linear contrast for iDPC), the lighter oxygen columns are more clearly visualized in the iDPC micrograph.^43^ The DFT-optimized *Imma* crystal structure model, overlaid across both the HAADF and iDPC images, shows excellent agreement with the experimentally observed atomic arrangement, providing compelling visual confirmation of the structural model.

**Figure 5:**
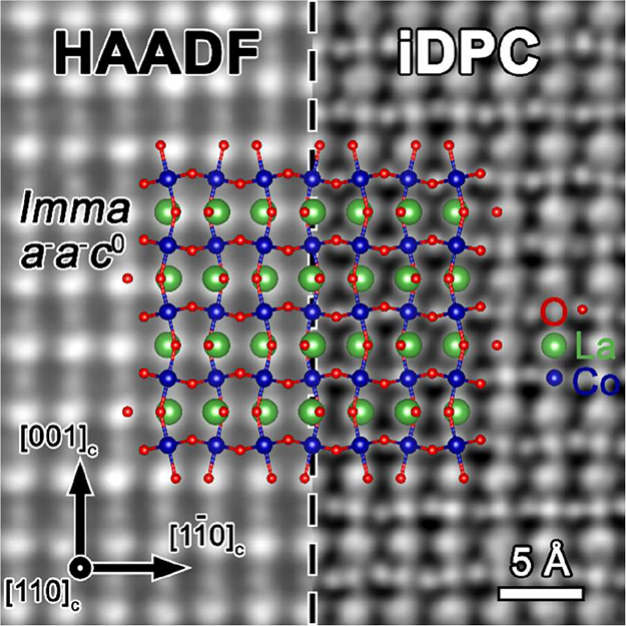
Composite HAADF (left) and iDPC (right) micrographs of the LaCoO_3_ (35 nm) film on the SrTiO_3_(001) substrate, viewed along the [110]_c_-zone axis. The HAADF and iDPC images were acquired simultaneously from the same region; the left half displays the HAADF signal and the right half the iDPC signal. A simulated *Imma* LaCoO_3_ structural model, corresponding to the DFT-optimized *a* ^–^ *a* ^–^ *c* ^0^ oxygen octahedral tilting system, is overlaid across both panels, with La, Co, and O atoms represented by green, blue, and red spheres, respectively.

The presence of ferromagnetism or any other type of cooperative magnetism in LaCoO_3_ films requires a non-negligible concentration of magnetic ions at low temperatures. Such behavior differs from bulk LaCoO_3_ where the ground state is made up almost exclusively nonmagnetic LS Co^3+^ (*S* = 0) ions. The magnetic ions could be HS Co^3+^ (*S* = 2), IS Co^3+^ (*S* = 1), or if oxygen vacancies are present HS Co^2+^ (*S* = 3/2). To investigate this aspect directly, synchrotron X-ray absorption spectra (XAS) at the Co L_2_ and L_3_ edges were collected at 36 K for 10 nm films grown on both SrTiO_3_ and LaAlO_3_. In bulk samples spectra collected at this temperature are dominated by LS Co^3+^ at this temperature, so the detection of other spin states and/or oxidation states can be directly attributed to the effects of strain or defects, or a combination of the two.

Figure 6a compares XAS measurements on LaCoO_3_ films grown on both SrTiO_3_ and LaAlO_3_ with several reference spectra where the spin state and oxidation state are well-defined.^17^ HS Co^3+^ has a distinctive pre-edge peak, labeled A, while LS Co^3+^ has a distinctive postedge feature labeled by peak B. The peak located near 777 eV (labeled C) is a pre-edge feature associated with HS Co^2+^. Notably, no features that can be attributed to Co^2+^ are seen in either film, further supporting our assertion that the concentration of oxygen vacancies is minimal. While the spectra for the films grown on SrTiO_3_ and LaAlO_3_ are similar, there are clear differences, as highlighted in the top panel of Figure 6b. The film grown on LaAlO_3_ has a pronounced shoulder on the high-energy side of the L_3_ peak, as would be expected if LS Co^3+^ is the dominant spin state. The film grown on SrTiO_3_ has a less prominent shoulder on the high energy side and greater intensity on the low-energy side of the peak. Both features that would be expected if some LS Co^3+^ ions are converted into HS Co^3+^.

**Figure 6:**
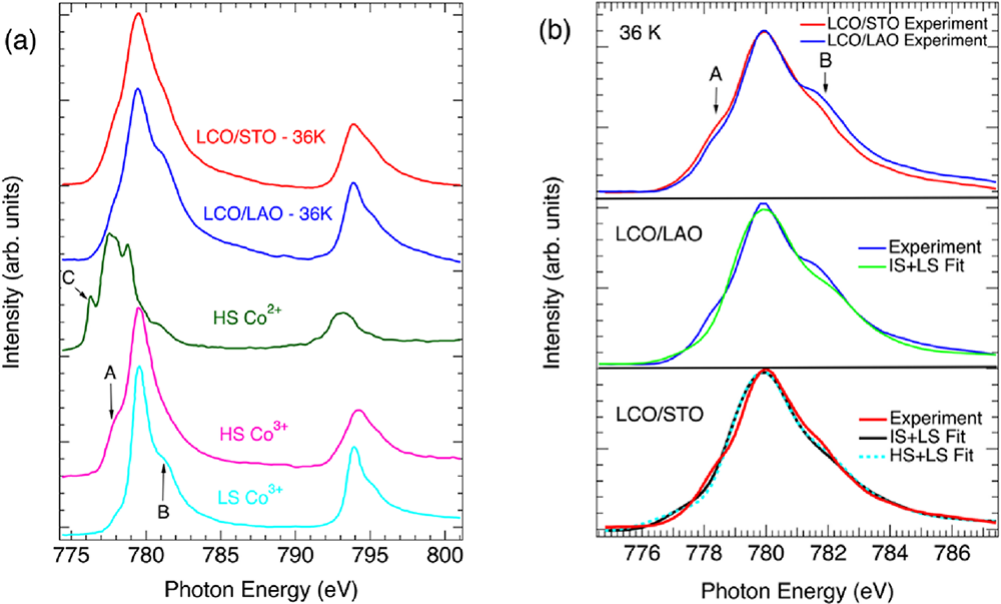
X-ray absorption spectra for (a) the Co L_2_ and L_3_ edges for various Co-containing oxides, taken from ref 17. The A label marks the pronounced low energy shoulder for HS Co^3+^, B marks the high-energy shoulder for LS Co^3+^, and C marks the lowest energy feature for Co^2+^. (b) The Co L_3_ XAS spectra taken at 36 K for LaCoO_3_ films grown on SrTiO_3_ and LaAlO_3_. Simulated fits to the data for a LaCoO_3_ film on LaAlO_3_ and SrTiO_3_. Details of the fits are given in the text.

In the bottom two panels of Figure 6b the experimental XAS data for these films are fit to simulations obtained from multiplet theory calculations.^44,45^ The fits were obtained by taking a linear combination of LS Co^3+^ and either HS Co^3+^ or IS Co^3+^ spectra. These simulations were produced using CTM4XAS and an 80% Slater integral reduction with 10Dq = 2.0 eV for the HS and IS states and 2.2 eV for the LS state. The IS state is differentiated from the HS state by applying additional crystal field parameters associated with *D*_4h_ symmetry, *D*_t_ = 0.04 eV and *D*_s_ = 0.07 eV. The resulting simulations were broadened using a Lorentzian-Fano line shape and slightly energy shifted to match the experimental spectra.

The fitting indicates that the films grown on LaAlO_3_ contain predominantly LS Co^3+^ and thus are relatively insensitive to whether the magnetic ions are modeled as HS or IS Co^3+^. When fit assuming a mixture of LS and HS ions the modeling indicates that the films contain 98(5)% LS Co^3+^ and 2(5)% HS Co^3+^. When fit assuming a mixture of LS and IS the numbers change slightly to 95(6)% LS Co^3+^ and 5(6)% IS Co^3+^. In either case, the concentration of magnetic Co^3+^ ions is within one standard deviation from zero, and the fits are indistinguishable.

The fitting yields quite different results for LaCoO_3_ grown on SrTiO_3_. If we assume a mixture of LS and IS ions, as might be stabilized by the axial compression of the octahedra revealed by the DFT calculations, the best fit is obtained for a mixture of 62(6)% LS Co^3+^ and 38(6)% IS Co^3+^. If we fit the spectrum with a mixture of LS and HS Co^3+^ ions the concentration of magnetic HS Co^3+^ ions is reduced to 27(6)%, with a comparable quality of fit. Within the constrained variable approach used to fit the spectra, it is not possible to definitively say whether the film contains a mixture of LS and HS ions, a mixture of LS and IS ions, or possibly a mixture of all three spin states. However, we can say with certainty that films grown on SrTiO_3_ contain a non-negligible, but minority, fraction of either HS or IS ions, unlike films grown on LaAlO_3_.

Simple logic dictates that a mixture of spin states is needed to stabilize an insulating ferromagnetic ground state. If all Co^3+^ ions were to adopt a HS state the superexchange interactions between neighboring ions would be antiferromagnetic and the magnetic structure would be a G-type antiferromagnet, similar to LaFeO_3_. If the Co^3+^ ions were exclusively in an IS state, various pattens of orbital ordering could lead to some degree of local ferromagnetic coupling, as seen in LaMnO_3_, but an overall antiferromagnetic ground state is still the most likely outcome. Fujioka et al. proposed a ferrimagnetic arrangement of IS and HS Co^3+^ in a 3:1 ratio for films grown on LSAT substrates (*T*_C_ = 94 K, *M*_sat_ ≈ 0.7 μ_B_/Co).^46^ While we cannot rule out that such a model could be applicable to the films they studied, the absence of LS Co^3+^ assumed in their model is clearly inconsistent with the XAS data collected here. Even a rock salt ordering of a 1:1 mixture of LS and HS Co^3+^ ions would be expected to stabilize an antiferromagnetic ground state, as seen for double perovskites like Sr_2_CoWO_6_.^47^ Therefore, an ordered pattern of HS and LS ions, where the magnetic HS ions make up <50% of the total Co^3+^ ions, seems the most promising way to stabilize an insulating ferromagnetic ground state. The challenge going forward is to identify the pattern of spin-state ordering that is consistent with ferromagnetic ordering, either by employing experimental probes to determine the arrangement of spin states at low temperatures and/or computational studies with large supercells that permit complex patterns of spin-state ordering.

The observation of a saturation magnetization on the order of ∼0.3 μ_B_ raises some doubts about the assumption often made in the literature that the ground state is a collinear ferromagnet. A HS Co^3+^ ion would have 4 unpaired electrons a local moment that is ∼4 μ_B_. If all such moments were to adopt a colinear, ferromagnetic arrangement, the fraction of HS Co^3+^ ions would need to be <10% of the total number of cobalt ions to give saturated magnetization values comparable to those observed in this study. It is hard to believe that such a dilute array of magnetic ions would have sufficiently strong superexchange coupling to stabilize magnetic ordering at temperatures ranging from 60–80 K. This suggests the magnetic ground state may be more complicated than a simple collinear ferromagnet. Possibilities include noncollinear magnetism, ferrimagnetism, or inhomogeneous magnetism, although the latter would seem to be inconsistent with our magnetic and structural data that shows little dependence on film thickness.

## 4 Conclusions

Epitaxial LaCoO_3_ films with thicknesses varying from 10 to 80 nm have been deposited using off-axis RF-sputtering. Ordered planes of oxygen vacancies, commonly observed in PLD grown films, are not observed in high-resolution TEM images, and no sign of Co^2+^ is seen in XAS spectra, confirming that the composition of these films approaches the ideal LaCoO_3_ stoichiometry. The effect of tensile strain on the structure is to break the rhombohedral symmetry and distort the symmetric octahedra of bulk LaCoO_3_. Films grown on SrTiO_3_ substrates exhibit *a* ^–^ *a* ^–^ *c* ^0^ octahedral tilting, orthorhombic *Imma* symmetry, and an axial compression of the Co-centered octahedra. XAS measurements performed at low temperature (36 K) show that films grown on SrTiO_3_ have a mixture of HS and LS Co^3+^ ions or IS and LS Co^3+^ ions. In contrast, films grown on LaAlO_3_ contain little if any HS Co^3+^ (similar to bulk LaCoO_3_). The strain-induced change in the distribution of spin states explains in part why tensile strain stabilizes long-range magnetic order.

While the observation of low temperature ferromagnetism on films grown on SrTiO_3_ substrates is consistent with earlier studies of PLD-grown films, the saturation magnetization of the films studied here, ∼0.3 μ_B_/Co, is smaller than most literature reports by a factor of ∼3. This reduction in magnetic moment is significant for two reasons. First, it infers that strain and oxygen vacancies work synergistically to enhance the ferromagnetism observed in many LaCoO_3_ thin films. Second, the size of the moment found here is inconsistent with a simple ferromagnetic alignment of the moments on the HS Co^3+^ ions, implying a more complicated low temperature magnetic structure.

## Supplementary Material



## Notes

Review of literature reports of the magnetic properties of tensile strained LaCoO_3_ films; powder XRD for LaCoO_3_ sputter target; out-of-plane lattice parameters measured by XRD for films of varying thickness grown on SrTiO_3_ and LaAlO_3_ substrates; reciprocal space mapping of LaCoO_3_ films; electrical resistance as a function of temperature; magnetic measurements on films grown on LaAlO_3_ substrates; a summary of magnetic properties measured for LaCoO_3_ films grown on SrTiO_3_ substrates; and the crystallographic details of the DFT-optimized structure of LaCoO_3_ grown on SrTiO_3_ (PDF)
