# Supplementary material for: Understanding the Effects of Tensile Strain on the Structure and Magnetism of Stoichiometric LaCoO3 Films
Source: Chem Mater. 2026 Mar 12;38(6):2904–12. doi: 10.1021/acs.chemmater.5c03290 (PMC13019621; doi:10.1021/acs.chemmater.5c03290)
Supplement: Supplementary file 1 [file cm5c03290_si_001.pdf]

Understanding the Effects of Tensile Strain on the Structure and Magnetism of Stoichiometric $LaCoO_3$ Films

Daniel Russell,[1] Rebecca M. Haight,[2] Binzhi Liu,[3] Ali Barooni,[3] Allen Partin,[2] Alevtina Smekhova,[4] Florian Kronast,[4] L. Robert Baker,[2] Maryam Ghazisaeidi,[3] Jinwoo Hwang,[3] Fengyuan Yang,[2] Patrick M. Woodward[1,2 *]

[1] Department of Physics, The Ohio State University, 191 W. Woodruff Avenue, Columbus, Ohio 43210, United States

[2] Department of Chemistry and Biochemistry, The Ohio State University, 100 W. 18th Avenue, Columbus, Ohio 43210, United States

[3] Department of Materials Science and Engineering, The Ohio State University, 140 W. 19th Avenue, Columbus, Ohio 43210, United States

[4] Helmholtz-Zentrum Berlin für Materialien und Energie, Albert-Einstein-Strasse 15, 12489 Berlin, Germany

* Corresponding Author:

PMW: woodward.55@osu.edu

Table S1: A summary of the magnetic properties of $LaCoO_3$ films reported in the literature. All films are grown under tensile strain, either on $SrTiO_3$ or $(La_{0.3}Sr_{0.7})(Al_{0.65}Ta_{0.35})O_3$ (LSAT) substrates.

T_C (K)	M_{sat} (μ_B)	H_C (kOe)	Substrate	Thickness (nm)	Co^{2+} present	Reference
85 K	0.5 μ_B	7.5 kOe	LSAT	200 nm	N/A	Fuchs (2007)[9]
85 K	0.5 μ_B	7.5 kOe	$SrTiO_3$	70 nm	Yes	Mehta (2015)[11]
84 K	1 μ_B	5 kOe	LSAT	70 nm	Yes	Mehta (2015)[11]
80 K	0.5 μ_B	5 kOe	$SrTiO_3$	5 nm	N/A	Yoon (2021)[14]
85 K	2 μ_B	5 kOe	LSAT	14 nm	No	Guo (2019)[28]
85 K	1.2 μ_B	5 kOe	$SrTiO_3$	14 nm	No	Guo (2019)[28]
75 K	1 μ_B	3 kOe	$SrTiO_3$	12 nm	Yes	Chen (2023)[18]
80 K	1 μ_B	5 kOe	$SrTiO_3$	10 nm	Yes	Li (2023)[17]
83 K	1 μ_B	5 kOe	LSAT	15 nm	N/A	Chaturvedi (2020)[23]
78 K	1 μ_B	5 kOe	$SrTiO_3$	15 nm	N/A	Chaturvedi (2020)[23]
80 K	1 μ_B	5 kOe	LSAT	30 nm	No	Huang (2023)[19]

The $LaCoO_3$ powder used to make the sputtering target was synthesized using conventional solid-state synthesis from La_2O_3 and Co_3O_4. The purity of the black powder was confirmed using X-ray powder diffraction, shown in Figure S1. Only $LaCoO_3$ is observed and it possesses the expected structure which is distorted from cubic (space group $Pm\bar{3}m$) to rhombohedral (space group $R\bar{3}c$) by octahedral tilting. This powder was used as the target for the growth of stoichiometric thin films.

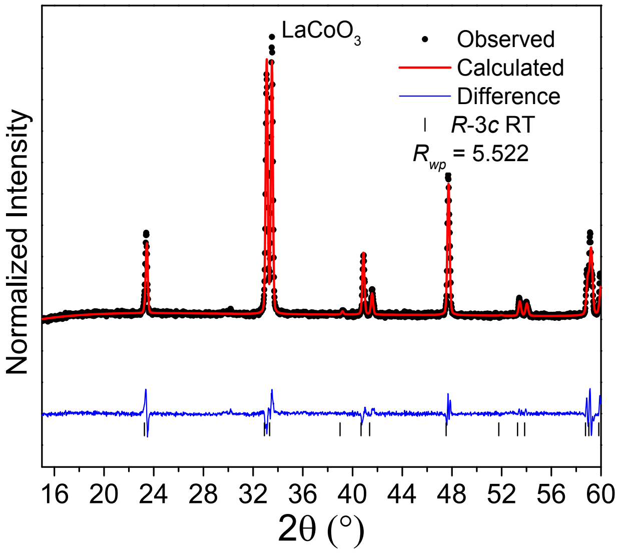

Figure S1: Rietveld refinements of the $LaCoO_3$ powder showed a pure, single-phase product.

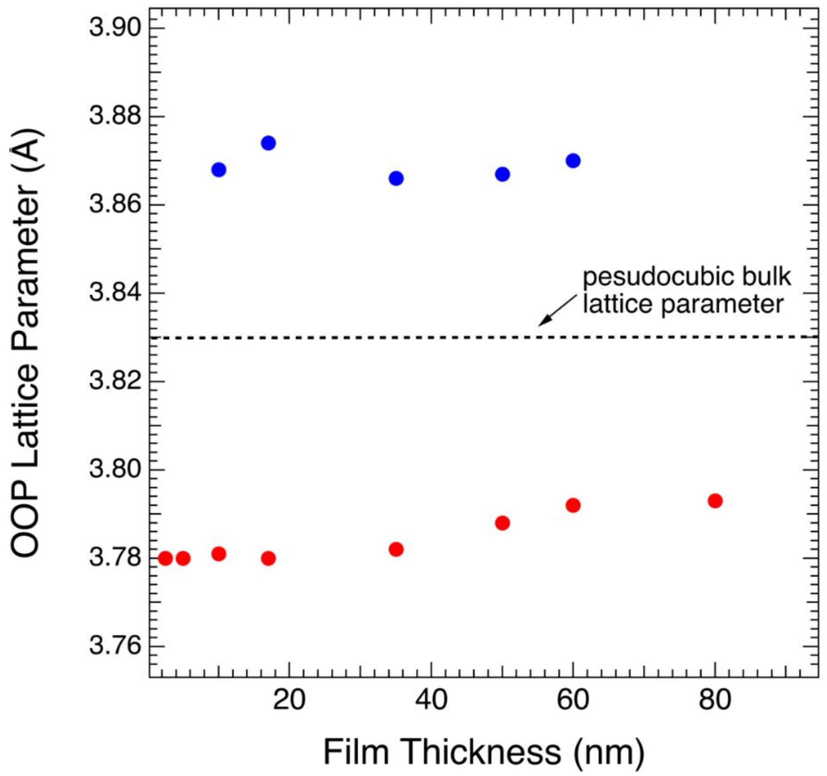

Figure S2: The out-of-plane (OOP) lattice parameters for $LaCoO_3$ films of various thicknesses grown on either under tensile strain on $SrTiO_3(001)$ or compressive strain on $LaAlO_3(001)$.

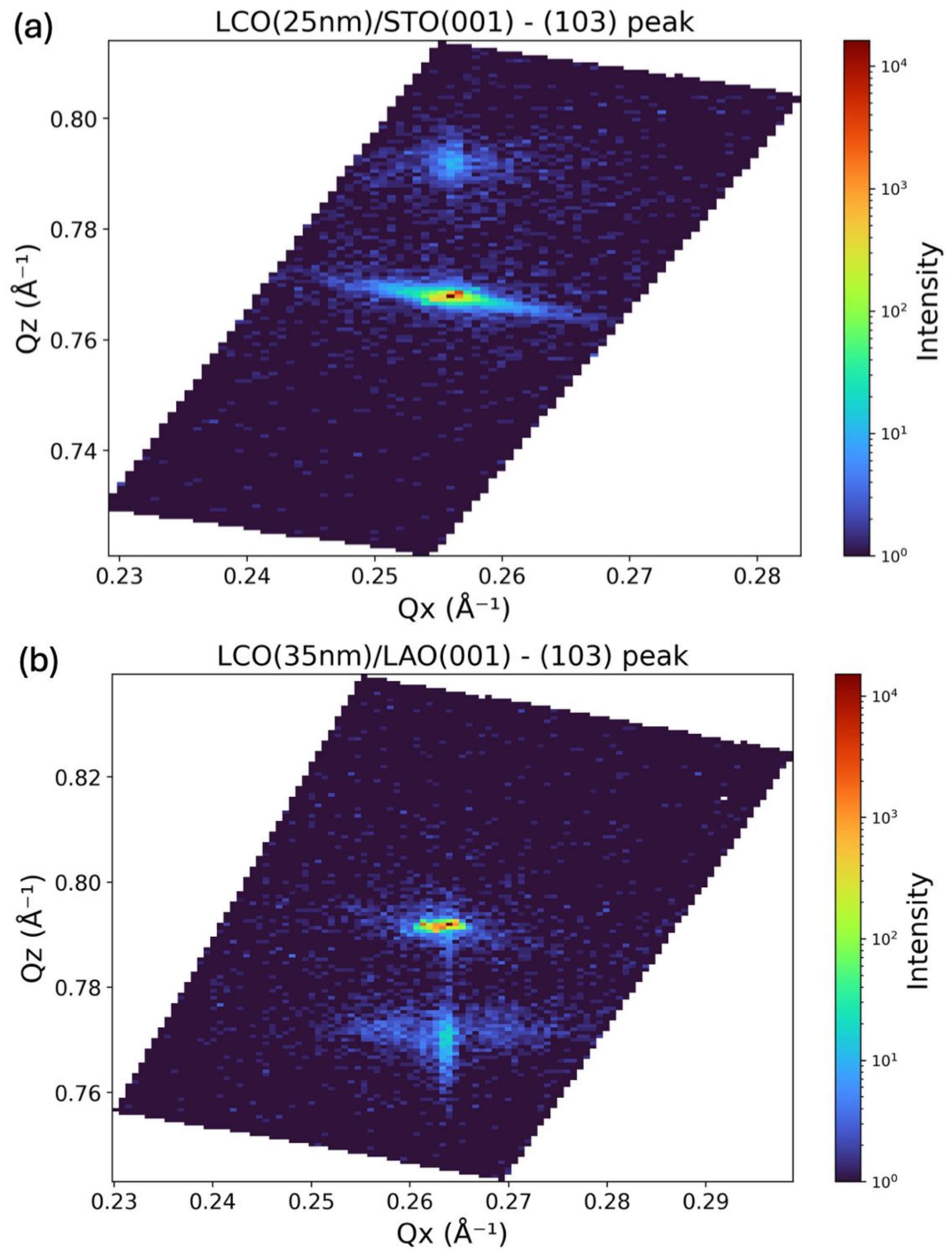

Figure S3: Reciprocal space mapping scans along the [103] axis of (a) the 25 nm thick $LaCoO_3$ film grown on $SrTiO_3$(001), and (b) the 35 nm thick $LaCoO_3$ film grown on $LaAlO_3$.

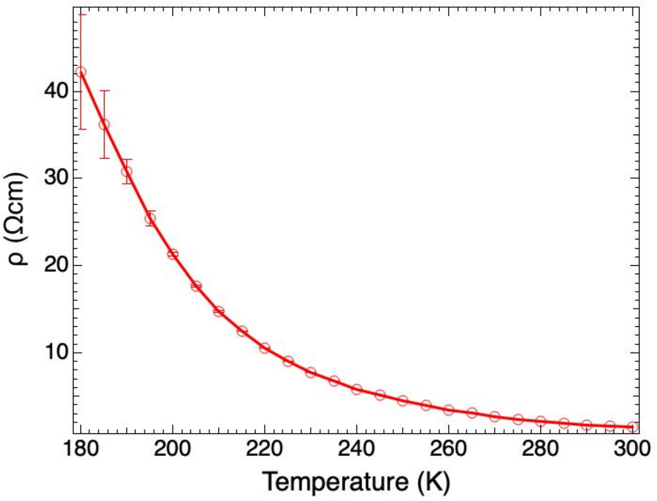

Figure S4: Resistivity as a function of temperature for a 20 nm $LaCoO_3$ film grown on a $SrTiO_3$(001) substrate.

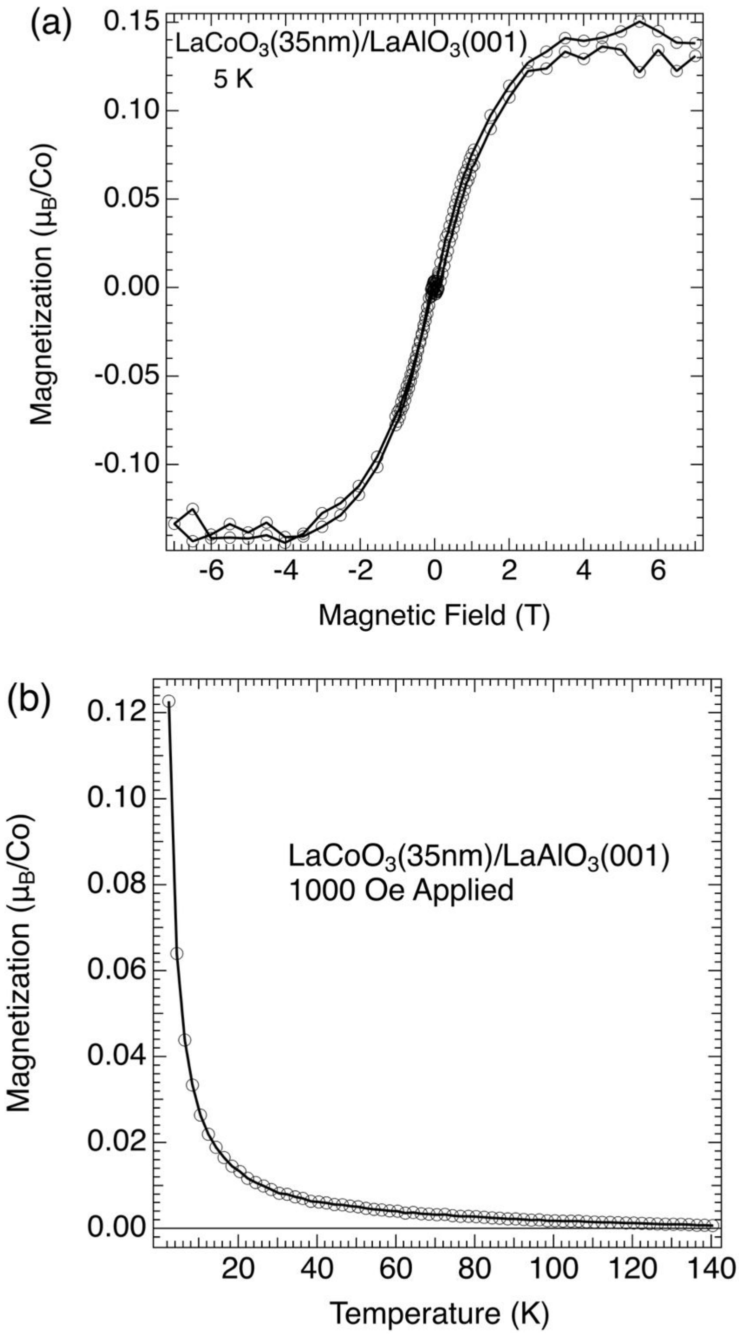

Figure S5: (a) Magnetization vs applied field for a $LaCoO_3$(35nm)/$LaAlO_3$(001) film. (b) Magnetization vs temperature for the same film.

Table S2: A summary of magnetic data for the films grown on $SrTiO_3$(001). No ferromagnetic behavior was observed for films grown on $LaAlO_3$.

**Thickness (nm)**	**T_C (K)**	**M_{sat} (μ_B/Co)**	**Coercivity (kOe)**
10	63	0.33	6
17	63	0.33	5
35	64	0.26	6
50	67	0.36	8
60	70	0.30	10
80	70	0.32	9

Table S3: The crystallographic coordinates for the DFT calculated, geometry optimized structure of $LaCoO_3$ constrained to lattice match with a $SrTiO_3$ substrate. To keep the c-axis perpendicular to the substrate the nonstandard *Ibmm* setting of space group #74 is used with lattice parameters a = 5.522 Å, b = 5.522 Å, and c = 7.511 Å. To convert to the standard *Imma* setting xyz → yzx.

**Atom**	**Wyckoff Site**	**x**	**y**	**z**
La	4e	0.500	0	¼
Co	4a	0	0	0
O(1)	4e	0.0656	0	¼
O(2)	8g	¼	−0.0345	¼
